# Research Resources: Comparative MicroRNA Profiles in Human Corona Radiata Cells and Cumulus Oophorus Cells Detected by Next-Generation Small RNA Sequencing

**DOI:** 10.1371/journal.pone.0106706

**Published:** 2014-09-04

**Authors:** Xian-Hong Tong, Bo Xu, Yuan-Wei Zhang, Yu-Sheng Liu, Chun-Hong Ma

**Affiliations:** 1 Institute of Immunology, Medical College of Shandong University, Ji'nan, China; 2 Center for Reproductive Medicine, Anhui Provincial Hospital Affiliated with Anhui Medical University, Hefei, China; 3 Hefei National Laboratory for Physical Sciences at Microscale and School of Life Sciences, University of Science and Technology of China, Hefei, China; Huazhong University of Science and Technology, China

## Abstract

During folliculogenesis, cumulus cells surrounding the oocyte differentiate into corona radiata cells (CRCs) and cumulus oophorus cells (COCs), which are involved in gonadal steroidogenesis and the development of germ cells. Several studies suggested that microRNAs (miRNAs) play an important regulatory role at the post-transcriptional level in cumulus cells. However, comparative miRNA profiles and associated processes in human CRCs and COCs have not been reported before. In this study, miRNA profiles were obtained from CRCs and COCs using next generation sequencing in women undergoing controlled ovarian stimulation for IVF. A total of 785 and 799 annotated miRNAs were identified in CRCs and COCs, while high expression levels of six novel miRNAs were detected both in CRCs and in COCs. In addition, different expression patterns in CRCs and COCs were detected in 72 annotated miRNAs. To confirm the miRNA profile in COCs and CRCs, quantitative real-time PCR was used to validate the expression of annotated miRNAs, differentially expressed miRNAs, and novel miRNAs. The miRNAs in the let-7 family were found to be involved in the regulation of a broad range of biological processes in both cumulus cell populations, which was accompanied by a large amount of miRNA editing. Bioinformatics analysis showed that amino acid and energy metabolism were targeted significantly by miRNAs that were differentially expressed between CRCs and COCs. Our work extends the current knowledge of the regulatory role of miRNAs and their targeted pathways in folliculogenesis, and provides novel candidates for molecular biomarkers in the research of female infertility.

## Introduction

Ovarian follicles, which are a densely packed shell of granulosa cells that contain an immature or mature oocyte, are ultimately responsible for the development, maturation, and release of a mature egg for fertilisation. In addition, they are also responsible for synthesising and secreting hormones that are essential for follicular development, the maintenance of the reproductive tract and menstrual cycle, and the development of female secondary sex characteristics [1].

During folliculogenesis, the granulosa cells differentiate into mural granulosa and cumulus cells where they perform cell-specific tasks [2], [3]. These cumulus cells derive from the same population of early follicles, but differentiate into two distinct groups of cells: 1) Those cells that lie directly on the zona pellucida are composed of the so-called “corona radiata cells”. Corona radiata cells (CRCs) are arranged radially around the oocyte and form about a two to three cell-thick layer. CRCs are connected to the oocyte via transzonal cytoplasmic projections until ovulation. These cellular projections allow the oocyte and cells to exchange information and metabolites [2]–[4]. Even after fertilisation, some of the CRCs can still retract with the oocyte without losing contract [4], [5]. 2) The other group of cells surrounds the CRCs and consists of more numerous cells, forming the so-called “cumulus oophorus cells (COCs)”, which are held together in a gelatinous matrix of hyaluronic acid. CRCs and COCs surround the oocyte both in the follicle and after ovulation, and they project into the antrum since secondary follicles [5]–[7]. The close interactive and mutual relationship between the oocyte, CRCs and COCs supports the follicular development and maturation of oocytes via sterol biosynthesis, the regulation of meiosis, gene transcription, and by protecting the oocytes [8]–[10].

Recently, a type of post-transcriptional regulator, microRNA (miRNA), has received wide-spread attention in ovarian granulosa cells during folliculogenesis [11]–[13]. miRNAs are endogenous non-coding RNAs. They average 21 nucleotides in size and function in the regulation of mRNA metabolism mainly via direct base-pairing interactions at the post-transcriptional level in a number of processes, including development, cancer, and stress responses. For instance, the conditional inactivation of Dicer1 (a ribonuclease required for miRNA production) in follicular granulosa cells by using the Amhr2-cre and Dicer1-loxp system led to an increased primordial follicle pool endowment, accelerated early follicle recruitment, and more follicle degeneration [14], [15]. A mutation in the hypomorphic Dicer allele (Dicer d/d) leads to female infertility due to impaired corpus luteum (CL) function. In addition, several studies have reported that some individual miRNAs participate in human folliculogenesis [16]–[17]. For example, miR-21 regulates the synthesis of COL4A1, which is a component of the basement membrane surrounding granulosa cells and the extracellular structure [18]. MiR-383 promotes steroidogenesis by targeting RBMS1 via the inactivation of c-Myc [19]. These studies suggest that miRNAs are involved in the regulation of granulosa cell-related biological processes during folliculogenesis and emphasise the importance of the comparative identification of the miRNA profiles in CRCs and COCs [20]–[23].

The development of next-generation sequencing (NGS) techniques has facilitated and improved the identification of miRNAs due to their high sensitivity [24]. Although high-throughput miRNA profiling has been carried out in ovarian somatic cells [25]–[27], comparative miRNA expression profiling of CRCs and COCs has not yet been conducted.

In this study, we determined the miRNA expression profiles, via NGS technology, of CRCs and COCs in order to characterise the ensemble of both known and novel miRNAs expressed in these cells. Moreover, GO and pathway analysis of the potential miRNA target genes for the differentially expressed miRNAs between CRCs and COCs indicated that miRNAs are involved in many important processes in human ovarian CRCs and COCs, including amino acid metabolism, glycolysis and cholesterol biosynthesis. Our results suggest that miRNAs in cumulus cells play an important role in oocyte maturation and ovarian follicular development, and this study provides a useful resource for the development of prophylactic strategies for female infertility.

## Materials and Methods

### Ethics Statement

The samples used for this study were collected from the Centre for Reproductive Medicine of Anhui Provincial Hospital Affiliated with Anhui Medical University, and this study was approved by the Ethics Committees on Human Research of Anhui Provincial Hospital Affiliated with Anhui Medical University (Approve ID: 20131357). The recruitment of patients was performed among infertile couples coming for ICSI-ET treatment at the Centre for Reproductive Medicine, Provincial Hospital, Anhui, between October 2012 and March 2013. All couples that agreed to participate in this study and all samples were obtained with written informed consent from all participants involved in the study.

### Patient Population and stimulation protocol

Five women from the Reproductive Medical Centre of Anhui Provincial Hospital, aged 29.1±2.7 (Mean±SD), undergoing ICSI-ET with a standard long stimulation protocol due to male factor infertility and achieving a clinical pregnancy were enrolled. All five patients were stimulated with the standard long gonadotropin-releasing hormone agonist (GnRHa, Diphereline; Ipsen Pharma. Biotech, Signes, France) protocol combined with the administration of recombinant FSH (Gonal-F, Merck Serono SA, Geneva, Switzerland). For oocyte retrieval, all patients underwent ovarian puncture (OPU) of follicles >15 mm after 36 h of administration of 10 000 IU human chorionic gonadotropin (hCG, LiZhu Pharma, ZhuHai, China). Only the cumulus–oocyte complexes with metaphase II (MII) oocytes were included in this study.

### Isolation of corona radiata cells and cumulus oophorus cells

The cumulus oophorus cells were collected and processed as previously described [28]. Briefly, the cumulus–oocyte complexes were retrieved 36 h after hCG treatment and washed in multiple dishes with flushing medium (William A. Cook Australia Pty. Ltd., Queensland, Australia). The cumulus oophorus cells were collected in fertilisation medium (William A. Cook Australia Pty. Ltd., Queensland, Australia) using two disposable needles and two 1-ml plastic disposable syringes without hyaluronidase. To avoid the interfusion of corona radiata cells, the innermost layers of cumulus oophorus cells were not collected. On the other hand, the corona radiata cells were collected as described in our previous publication and other reports [26], [28]. The corona radiata cells were separated from the oocyte by gentle pipetting with a 135-mm-diameter stripper pipette and micromanipulator system. MII oocytes were used for the ICSI procedure. The corona radiata cells and cumulus oophorus cells were pooled by centrifuging at 1500×g for 8 min and immediately frozen in liquid nitrogen until use.

### Library construction and sequencing

Library construction and sequencing was performed at BGI-Shenzhen. Briefly, for NGS analysis of miRNAs total RNAs were extracted from the human CRCs and COCs using TRIzol reagent (Invitrogen). These RNA samples were pooled from five patients and then subjected to 15% (w/v) denaturing PAGE, and the small RNA fragments of 18–28 nt were isolated from the total RNA and sequentially ligated to a pair of adaptors (5′adaptor-GTTCAGAGTTCTACAGTCCG-ACGATC, 3′ adaptor-TCGTATGCCGTCTTCTGCTTG). The small RNAs were reverse transcribed by reverse-transcription polymerase chain reaction (RT-PCR). Then, the purified RT-PCR products were sequenced by the Illumina Hiseq 2000 (Illumina, San Diego, CA, USA) according to Illumina's protocol.

### Computational analysis of sequencing data

The small RNA NGS data were analysed according our previously published tools using CPSS [29]. Briefly, after removing and trimming the adaptor sequences, filtering low quality reads and cleaning up contaminated reads, the occurrence of each unique read was counted as a tag, and these tags were mapped to the human genome using SOAP2.0 [30]. The known miRNAs were detected from the mapped tags by aligning them to miRBase, and the whole expression profiles of known miRNAs were presented as volcano plots. Other small RNAs were also detected by CPSS (all the reference datasets used for this study are the latest versions). MiRD and Mireap was used to predict novel miRNAs [24] (http://sourceforge.net/projects/mireap/), and the secondary structures of the potential miRNA precursors were predicted by RNA fold (http://rna.tbi.univie.ac.at/). All data obtained via NGS in this study are available in the ArrayExpress database (Accession number: E-MTAB-2264).

### Bioinformatics analysis for the miRNAs from CRCs and COCs

All of the bioinformatics analyses for the miRNAs in this study were performed according to the methods described in our previous reports [29]. To predict the miRNA targets, the targeted mRNA of differentially expressed and selected miRNAs were predicted by miRanda, Targetscan, and MicroCosm. All of the targeted genes predicted by any of these tools used for further analysis [31]–[33] followed the three rules: 1) Perfect match at the seed region (2–8 nt from the 5′ end of the miRNA); 2) the minimum free energy (MFE) of the miRNA/target duplex should be <−20 Kcal/mol; 3) the total score for an miRNA-mRNA pairs should be >140. For GO analysis of the predicted miRNA target genes from CRCs and COCs, the predicted target genes of differentially expressed and selected miRNAs were subjected to analysis of gene ontology terms [34]. The target genes were mapped to the GO annotation dataset, and the enriched biological processes were extracted using the hypergeometric test according our previous reports [29]. A GO term was identified as a key term in this study when its ratio of enrichment was >2 and the p-value was <0.05. For pathway analysis of the predicted miRNA target genes, the predicted miRNA targets were mapped to the signalling pathway annotation databases downloaded from KEGG [35]. The Fisher's exact test for hypergeometric distribution was used to detect the enriched pathway according our previous reports [29]. A relevant pathway was identified when the ratio of enrichment in this study was >1.5 and the p-value was <0.05.

### Expression detection by qRT-PCR

All quantitative real-time PCR analysis for the miRNAs in this study was performed according to the methods described in our previous reports [24]. The miRNA quantification was performed by quantitative real-time PCR using an Applied Biosystems StepOne Real-Time PCR System (Applied Biosystems, Foster City, California, USA) and a SYBR premix Ex Taq II kit (Takara) with the primers listed in the File S1. The snRNA level of U6 was used as an internal reference. The reactions were performed at 95°C for 30 s, followed by 40 cycles of 95°C for 5 s and 60°C for 31 s. All reactions were run at least in triplicate. In the experimental and control group, the PCR experiments were repeated four times with the pooled samples. Quantitative data from real-time PCR were compared using unpaired t-tests. P<0.05 was considered statistically significant.

## Results

### Overview of small RNA sequencing data

To determine the small RNA profile in human CRCs and COCs, we sequenced the small RNA libraries using Solexa NGS technology and acquired a total 15382469 and 15589634 raw reads from human CRCs and COCs. Thus, we removed the adaptor sequences and low quality reads, and 13021069 (CRCs) and 12900433 (COCs) clean reads remained (Table 1). The majority of these clean reads was 22 nt in length, with sizes varying between 18 and 26 nt. These clean reads were mapped to several filter databases, such as the Human Genome, tRNA, rRNA and Rfam sequence databases, and were subsequently mapped to miRbase (V14.1). After detecting other types of small RNAs, including rRNAs, repeats and snRNAs, 8534 unique tags corresponding to 9240863 reads in CRCs and 7577 unique tags corresponding to 9143970 reads in COCs were identified as known miRNAs (Table 1).

**Table 1:** The match results of clean reads from COCs and CRCs.

Small RNA category	COCs				CRCs			
	NO. of Unique tags	Percentage (%)	Total reads	Percentage (%)	NO. of Unique tags	Percentage (%)	Total reads	Percentage (%)
miRNA	7577	1.18	9143970	70.88	8534	1.39	9240863	70.97
piRNA	4987	0.77	137411	1.07	4942	0.81	144216	1.11
mRNA	145760	22.64	589264	4.57	135547	22.13	567979	4.36
rRNA	13186	2.05	245269	1.90	13566	2.22	180374	1.39
repeat	225720	35.06	1579160	12.24	232004	37.89	1700021	13.06
sRNA	10	0.00	10	0.00	37	0.01	58	0.00
snRNA	4082	0.63	297217	2.30	4008	0.65	150459	1.16
snoRNA	6161	0.96	62216	0.48	6877	1.12	103532	0.80
tRNA	5036	0.78	77440	0.60	6443	1.05	115197	0.88
Others	44726	6.95	188920	1.46	45061	7.36	193756	1.49
Unannotated	186563	28.98	579556	4.49	155361	25.37	624614	4.80
Total	643808	100	12900433	100	612380	100	13021069	100

### Chromosome location, expression level and enzymatic modification of the known miRNAs in human CRCs and COCs

**Figure 1:**
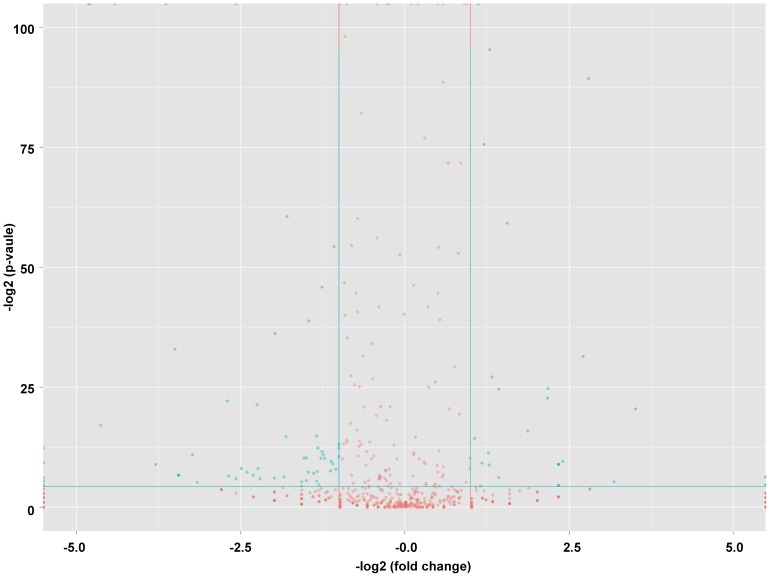
The miRNA expression profiles in CRCs and COCs are presented by volcano plots. The vertical lines correspond to 2-fold up- and down-regulation, respectively, and the horizontal line represents a p-value of 0.05. The red point in the plot represents the similarly expressed miRNAs without statistical significance, while the blue point in the plot represents the differentially expressed miRNAs with statistical significance.

**Figure 2:**
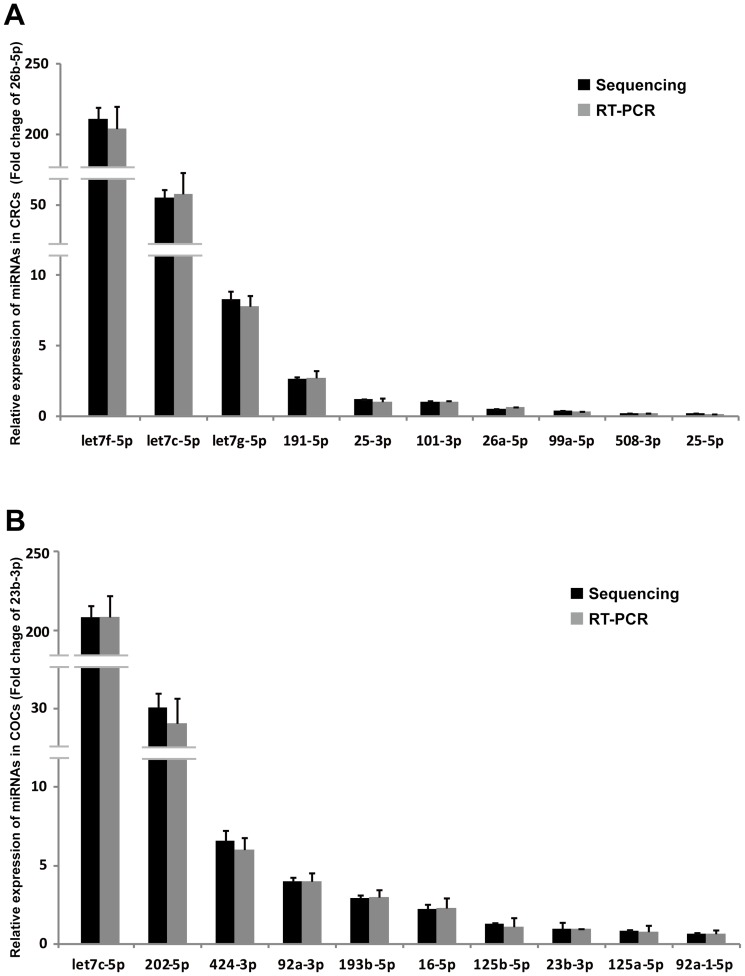
Confirmation of miRNA expression detected by NGS. Validation of the expression of ten miRNAs at different expression levels in CRCs (**A**) and COCs (**B**). In CRCs, the expression of miR-26b-5p was set as 1 and the expression levels of other miRNAs were compared with that of miR-26b-5p. In COCs, the expression of miR-23b-3p was set as 1 and the expression levels of other miRNAs were compared with that of miR-26b-5p.

**Figure 3:**
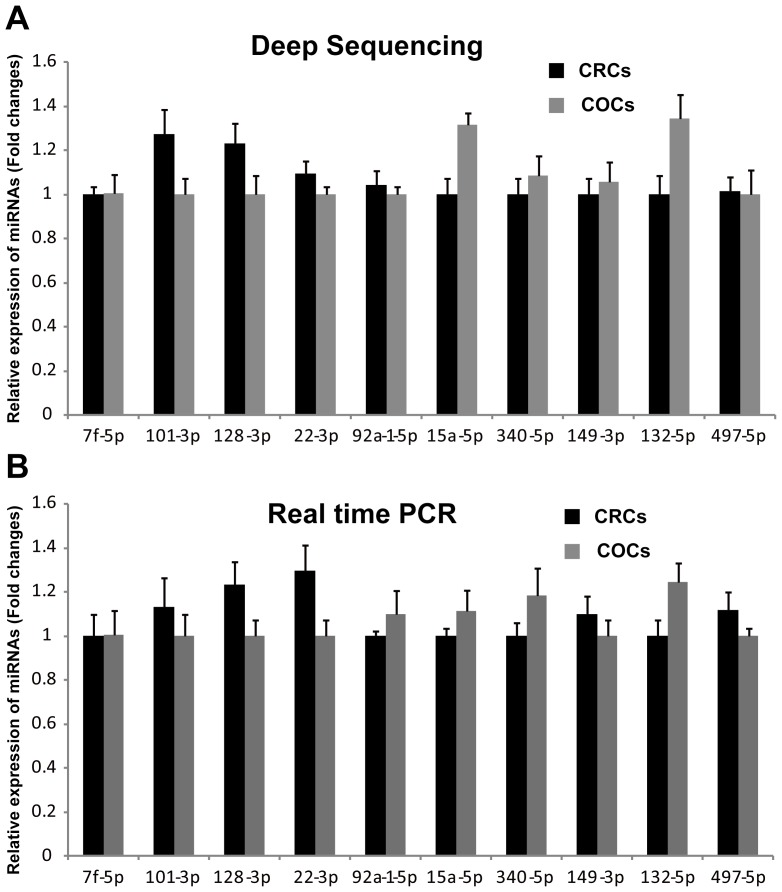
Confirmation of the expression of the most abundant miRNAs in both COCs and CRCs. Validation of the expression of ten miRNAs by NGS (**A**) and quantitative real-time PCR (**B**), demonstrating a similar expression pattern in COCs and CRCs.

The location of all clean reads and known miRNAs in different chromosomes were detected, and the distributions of reads and miRNAs are shown in Figure S1 and S2. In addition, the chromosome distributions of all clean reads and known miRNAs in CRCs and COCs were quite similar (Figure S1 and S2). Moreover, the expression profile of known miRNAs in CRCs and COCs were analysed, and most of the miRNAs were expressed equivalently (Figure 1, red spots). These results were consistent with the results obtained from CRCs and COCs that were derived from the same population of granulosa cells at the early follicle stage. The miRNAs in the let-7 family were clearly the most abundant miRNAs in both CRCs and COCs (Table 2), in which they participated in oocyte development and ovarian function [27]. To validate the miRNA expression detected by NGS, twenty known miRNAs in CRCs and COCs representing different levels of expression were randomly chosen for quantification by quantitative real-time PCR. The levels of these twenty known miRNAs measured by quantitative real-time PCR are consistent with the results obtained from NGS, which indicated that the expression of miRNAs detected by deep sequencing was reliable (Figure 2). In addition, the miRNAs with similar miRNA expression patterns in CRCs and COCs were also validated. The expression patterns of ten randomly chosen miRNAs were detected by quantitative real-time PCR in CRCs and COCs, and the results from two types of technology were also coincident (Figure 3). Recently, several reports have found that miRNAs exhibit post-transcriptional 5′ or 3′ end trimming, 5′ or 3′ end additions of nucleotides and nucleotide changes at different positions of the mature miRNA without a template [36]–[40]. These miRNA modifications and the miRNA editing may increase miRNA stability or strengthen miRNA-targeted mRNA interaction; these changes may even be involved in regulatory processes [41]. Thus, the miRNA modifications and editing of the known miRNAs in CRCs and COCs were identified (Table S1 and Table S2). Hsa-miR-320a showed the same modification at the 5′ end in both CRCs and COCs, indicating that miRNA-320a participates significantly in ovarian cumulus cell-related processes and functions [42] (Table S1). The let-7 family has been reported to be involved in the regulation of gestation, follicular development, oocyte growth and hormone response [27]. Thus, the miRNAs in the let-7 family showed most abundant expression in COCs and CRCs, with a significant amount of miRNA editing, suggesting that the diversification of miRNA editing and function of let-7 family members in CRCs and COCs might be involved in the processes of folliculogenesis and oocyte maturation (Table S2).

**Table 2:** Top 10 lists of most abundant miRNAs expressed both in COCs and CRCs.

miRNA name	COCs		CRCs	
	Absolute count	Relative count (Average rpm)	Absolute count	Relative count (Average rpm)
hsa-let-7f-5p	3366051	260925.43	3413634	262162.27
hsa-let-7a-5p	1945425	150803.08	1833006	140772.31
hsa-let-7b-5p	1333881	103398.16	1619389	124366.82
hsa-let-7c-5p	827627	64154.98	728943	55981.73
hsa-miR-320a	293263	22741.10	321620	24699.97
hsa-let-7e-5p	209116	16210.00	249482	19159.79
hsa-miR-3184-3p	153405	11889.21	170093	13049.01
hsa-miR-140-3p	130618	10125.09	131450	10095.02
hsa-let-7g-5p	96955	7515.64	93115	7151.10
hsa-let-7d-5p	43054	3337.41	45930	3527.36

Expression is presented as absolute reads and average reads per million reads (rpm).

### MicroRNAs differentially expressed in CRCs and COCs

**Figure 4:**
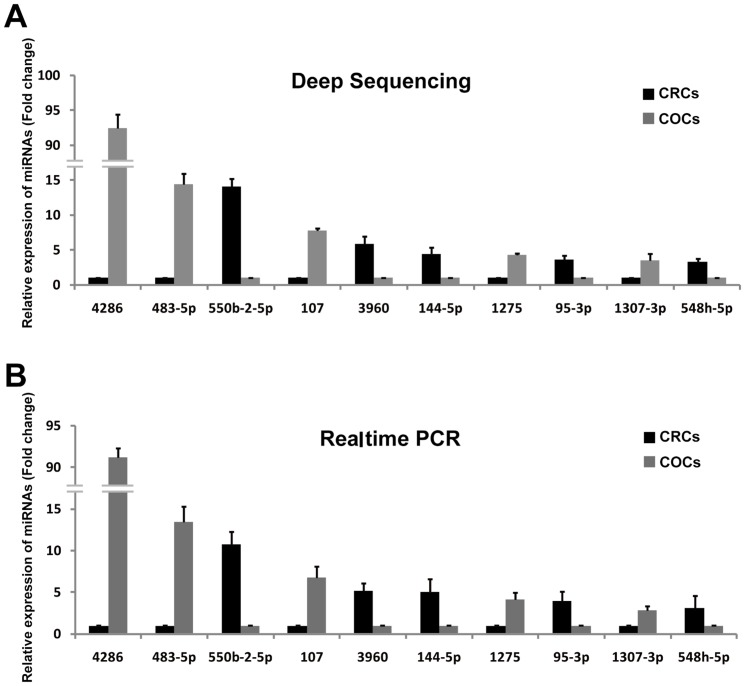
Confirmation of the differentially expressed miRNAs between COCs and CRCs. Validation of the expression of ten miRNAs by NGS (**A**) and quantitative real-time PCR (**B**), showing differentially expressed miRNAs in COCs and CRCs.

To detect known miRNAs that are differentially expressed in CRCs and COCs, the counts of each type of miRNA were first normalised based on the total number of all of the clean reads mapped onto the genome in CRCs or COCs (normalised counts are displayed as reads per million, RPM), and then compared between the CRCs and COCs. Therefore, 72 known miRNAs were differentially expressed between CRCs and COCs (fold change>2 and P<0.05), including 44-fold higher expression levels in CRCs and 28 in COCs (Table S3). To validate the expression of these differentially expressed miRNAs detected by NGS technology, 10 known miRNAs representing two types of expression patterns were randomly chosen for quantification by real-time PCR (Figure 4). The expression levels of ten differentially expressed miRNAs measured by quantitative real-time PCR were consistent with the results obtained from NGS technology, which indicated that the identification of differentially expressed miRNAs in this study was reliable (Figure 4).

### Prediction of the miRNA targeted genes and pathways

After the detection of a number of known miRNAs that were differentially expressed between CRCs and COCs, we then identified the targeted genes, signalling pathways and biological functions that could potentially be targeted by these miRNAs. The putative target genes of the differentially expressed miRNAs were predicted using miRanda, Targetscan, and MicroCosm [31]–[33] with the strict criteria described in the Materials and Methods (File S2). Therefore, we used GO and KEGG pathway analysis to enrich the involved biological pathways from the predicted targets (Table 3 and Table 4). After GO analysis, we found that the predicted targets of differentially expressed miRNAs appeared to be involved in a broad range of biological processes, with most of the targets related to protein metabolism and modification (e.g., protein demethylation, proline biosynthetic process and positive regulation of histone H3-K4 methylation), energy metabolism (e.g., response to ATP, cellular response to cholesterol, and coenzyme A biosynthetic process) and cell differentiation and regulation (e.g., CD4-positive, CD25-positive, alpha-beta regulatory T cell differentiation, hypothalamus cell differentiation and positive regulation of uterine smooth muscle contraction) (Table 3). Moreover, we enriched the biological pathway of predicted miRNA targeted genes by KEGG pathway analysis. Several signalling pathways were found to be involved, including amino acid metabolism (e.g., arginine, proline, alanine, aspartate and glutamate metabolism), common signalling pathways (e.g., Wnt signalling pathway), and cellular junctions (e.g., tight junctions) (Table 4).

**Table 3:** GO analysis for predicted targets of differentially expressed miRNAs between COCs and CRCs.

GO number	Go biological process	Targeted genes	Enrichment ratio	P vaule
GO:0015722	Canalicular bile acid transport	ABCB11 AQP8 AQP9 MIP	13.13	0.01
GO:0002361	CD4-positive, CD25-positive, alpha-beta regulatory T cell differentiation	FOXP3 FUT7 NCOR1	9.57	0.04
GO:0006482	Protein demethylation	KDM1A PHF2 PPME1	7.29	0.02
GO:0021979	Hypothalamus cell differentiation	OTP POU3F2 PROP1	6.57	0.02
GO:0001778	Response to ATP	DGKQ IL1B KCNJ11 PLCG2 SELL SLC8A1	5.26	0.03
GO:0006561	Proline biosynthetic process	ALDH18A1 ALDH4A1 PYCR1 PYCR2 PYCRL	4.69	0.01
GO:0071397	Cellular response to cholesterol	AACS INHBA INHBB LRP6	4.38	0.03
GO:0070474	Positive regulation of uterine smooth muscle contraction	ADRA2B LCK OXTR TACR3	4.22	0.03
GO:0051571	Positive regulation of histone H3-K4 methylation	BRCA1 DNMT1 DNMT3B PAXIP1	4.18	0.03
GO:0071436	Coenzyme A biosynthetic process	PANK1 PANK2 PANK3 PPCDC	3.36	0.02

**Table 4:** KEGG pathway analysis for predicted targets of differentially expressed miRNAs between COCs and CRCs.

Pathway name	Targeted genes	Enrichment ratio	P vaule
Fatty acid biosynthesis	PPA AOR ANG SEW	11.24	0.014
Glioma	MEK P21 ERK RAF EGF CDK4/6 E2F	9.66	0.022
AlAnine, Aspartate and Glutamate metabolism	GPT CPS1 IL4I1 CAD ABAT ASP5	9.65	0.017
Wnt signaling pathway	TAK1 NLK APC CAN WNT1 GBP ICAT	8.74	0.014
Pantothenate and CoA biosynthesis	PANK2 PPCDC	8.08	0.030
Arginine and proline metabolism	GDH2 ACT ARG56 PRODH2 CNDP1	7.32	0.026
Amoebiasis	IL6 COL CP PKA PLC IL12 CD14	7.16	0.029
Glycosphingolipid biosynthesis-LACto and NEO-lactoseries	FUT9 FUT7 B3GNTL2 B4GALT1	6.89	0.033
Basal cell carcinoma	APC FZD4 BMP HIP1 GLI1	5.29	0.028
Melanogenesis	PKA PLC SCF CBP DCT WNT	4.81	0.035
NON-SMAll Cell lung cancer	PI3K PDK1 EGFR BAD	4.15	0.037
Chagas disease	CCL3 CCL3L3 FASLG GNAL PLCB2	3.17	0.023

### Identification of Novel miRNAs and their targeted genes and pathways

**Figure 5:**
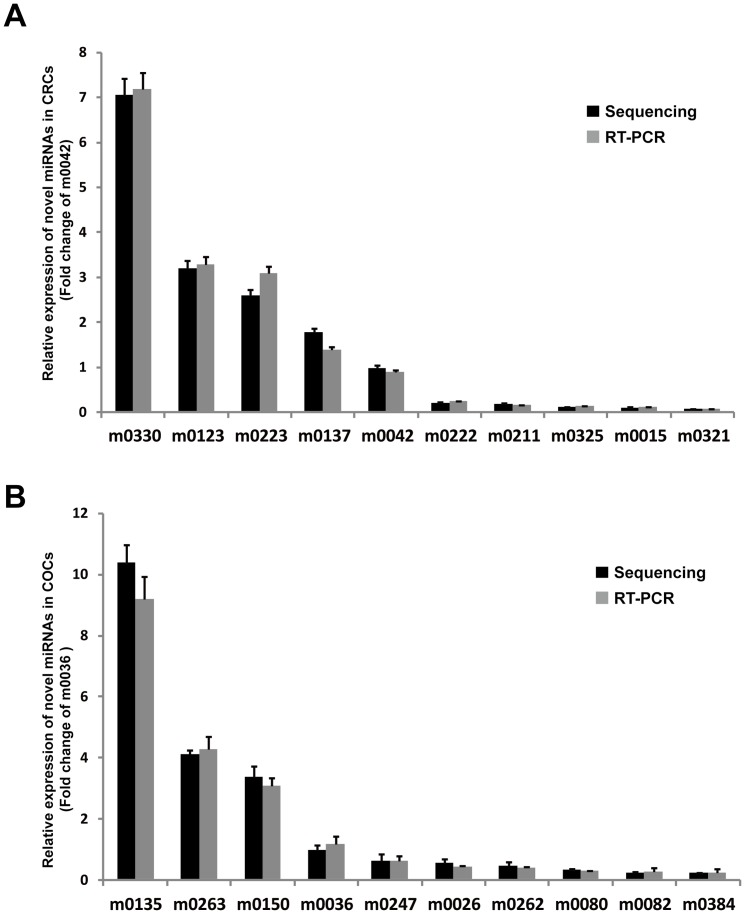
Confirmation of novel miRNA expression detected by NGS. Validation of the expression of ten novel miRNAs at different expression levels in CRCs (**A**) and COCs (**B**). In CRCs, the expression of novel miR-m0042 was set as 1 and the expression levels of other miRNAs were compared with that of novel miR-m0042. In COCs, the expression of novel miR-m0036 was set as 1 and the expression levels of other miRNAs were compared with that of novel miR-m0036.

The NGS techniques have revolutionised the identification of low expression or novel small RNAs with high levels of sensitivity and accuracy. Therefore, to detect more potential miRNAs in human CRCs and COCs, the unclassified reads were further processed using Mireap and MiRD (http://sourceforge.net/projects/mireap) [24]. Mireap and MiRD predicted the novel miRNAs based on default parameters with read counts greater than 45, which were defined as candidate novel mature miRNAs. Therefore, the novel miRNA genes encoding mature miRNAs were identified in CRCs and COCs (File S3), and the top ten most abundant novel miRNAs in CRCs and COCs are listed in Table 5 and Table 6. Notably, six of the same novel miRNAs were identified in both CRCs and COCs (Table 5 and Table 6, bold font), which was consistent with the known similarity of the miRNA expression profiles of CRCs and COCs (Figure 1). These novel miRNAs were confirmed by quantitative real-time PCR (Figure 5). Therefore, we predicted the targeted genes of novel miRNAs using miRanda, and these putative target genes for these identified novel miRNAs in CRCs and COCs were also assessed by GO and KEGG pathway analysis. The enrichment of targets according to GO analysis revealed that they appeared to be involved in a broad range of biological processes (Table S4 and Table S5). According to KEGG pathway analysis of these putative targets, several key pathways were enriched (Table S6 and Table S7).

**Table 5:** Novel miRNAs predicted from small RNA sequencing data of CRCs.

miRNA name	Mature sequence	Read counts	Location of novel miRNA precursor
m0330	TAGCAGCGGGAACAGTTCTGCAG	12020	chrX:133680351..133680433:-
**m0123**	**CCGGAGCTGGGGATTGTGGGT**	**5455**	**chr17:66015982..66016062:-**
**m0223**	**TGAGGTAGTAGTTTGTACAGTTT**	**4425**	**chr3:52302288..52302383:-**
**m0137**	**CACCCGTAGAACCGACCTTGCG**	**3033**	**chr19:52195861..52195939:+**
**m0042**	**AACCCGTAGATCCGAACTTGTGG**	**1700**	**chr11:122022932..122023014:-**
**m0222**	**CAACGGAATCCCAAAAGCAGCTGTT**	**369**	**chr3:49058053..49058137:-**
**m0211**	**TCGAGGACTGGTGGAAGGGCCTTT**	**336**	**chr2:219923403..219923479:-**
m0325	TTATAATACAACCTGATAAGTG	215	chrX:73507121..73507191:-
m0015	TTCCTATGCATATACTTCTTTGA	193	chr10:135061028..135061107:-
m0321	TGAGGTAGTAAGTTGTATTGTTG	152	chrX:53583191..53583291:-

**Table 6:** Novel miRNAs predicted from small RNA sequencing data of COCs.

miRNA name	Mature sequence	Read counts	Location of novle miRNA precursor
**m0135**	**CCGGAGCTGGGGATTGTGGGT**	**8795**	**chr17:66015982:66016062:-**
**m0263**	**TGAGGTAGTAGTTTGTACAGTTT**	**3508**	**chr3:52302288:52302383:-**
**m0150**	**CACCCGTAGAACCGACCTTGCG**	**2872**	**chr19:52195861:52195939:+**
**m0036**	**AACCCGTAGATCCGAACTTGTGG**	**848**	**chr11:122022932:122023014:-**
**m0247**	**TCGAGGACTGGTGGAAGGGCCTTT**	**548**	**chr2:219923403:219923479:-**
m0026	AAGACGGGAGGAAAGAAGGGAGTGG	495	chr11:2155360:2155442:-
**m0262**	**CAACGGAATCCCAAAAGCAGCTGT**	**410**	**chr3:49058055:49058137:-**
m0080	TGGTTTACCGTCCCACATACAT	301	chr14:101490127:101490200:+
m0082	TGTGACTGGTTGACCAGAGGGG	230	chr14:101521021:101521100:+
m0384	TGAGGTAGTAAGTTGTATTGTTGT	213	chrX:53583190:53583291:-

## Discussion

Folliculogenesis is a multi-faceted and tightly regulated process that includes primordial follicle assembly, follicle growth and atresia, and oocyte ovulation. The granulosa cells surrounding the oocytes play a major role in these processes [1]–[2]. Dysfunctional granulosa cells are associated with abnormal folliculogenesis, e.g., polycystic ovary syndrome (PCOS). However, to date, only few contributing factors have been detected to be involved in the dysfunction of follicular granulosa cells. Recently, an increased number of reports have indicated that ovarian granulosa cells are strictly regulated post-transcriptionally, while small RNAs are the key regulators at this level [11]–[17].

Generating expression profiles of small RNAs in human CRCs and COCs facilitates the understanding of their roles in the regulation of folliculogenesis. Although several differentially expressed miRNAs in CRCs and COCs were detected, the whole miRNA expression profiles were highly similar between the two cell types. This finding may be because both cell types are derived from the same population in the early follicles and participate in a large number of similar processes in support of oogenesis [43]–[44]. Consistent with the previous report, the most abundant miRNAs in both CRCs and COCs were those of the hsa-let-7 family [26], [27], [45]–[48], which has been reported to be involved in the regulation of gestation, follicular growth, ovarian cell steroidogenesis, development of ovarian cancer and hormone response [27], [45]–[48]. For example, hsa-let-7f has been described as a tumour suppressor in breast cancer cell lines and as a regulator controlling human ovarian cell steroidogenesis [45], [46]. Hsa-let-7b was also found to participate in follicular development in vitro and was found necessary for the normal development of the corpus luteum in mice [47], [48]. Another of the most abundant miRNAs, miR-320a, is expressed at much lower levels in the follicular fluid of PCOS patients and is also involved in the regulation of estradiol concentration [49]. All of these reports suggest that the post-transcriptional regulation of gene expression by miRNAs plays an important role in ovarian cumulus cells.

Moreover, in addition to determining the miRNA expression profile in CRCs and COCs, we were also interested in determining the differential miRNA profiles and their roles between the human CRCs and COCs. In total, 72 miRNAs were expressed differentially between human CRCs and COCs. Quantitative real-time PCR was used to validate these differentially expressed miRNAs, and it was shown that all tested miRNAs were differentially expressed in the two cell types. Thus, we conducted GO term annotation and KEGG pathway analysis for the identified miRNAs based on the prediction of miRNA targets. Notably, the metabolisms of several individual amino acids were enriched in the GO biological processes. Because oocytes are deficient in their ability to synthesise and transport several types of amino acids, the cumulus cells must provide oocytes with the amino acids or substrates for the metabolism of these amino acids [50]–[54]. For instance in mice, oocytes cannot directly synthesise some amino acids, such as L-alanine, and thus require that cumulus cells synthesise and transfer these amino acids into oocytes [50], [51]. Oocytes are connected to surrounding cumulus cells via membrane specialisations, such as gap junctions, which act as physical channels for the transport of metabolites and nutrition between the oocyte and the cumulus cells [50]–[54]. Obviously, the CRCs play a more important role in these gap junctions and the related amino acid metabolism and transport than do COCs because CRCs are arranged radically directly around the oocytes [28], [54], [55], [56]. In this study, the identified miRNAs, which were differentially expressed between CRCs and COCs, were found to participate in the regulation of amino acid metabolism. These results suggested that miRNAs may be involved in the bidirectional communication between oocytes and the regulation of amino acid metabolism in CRCs.

Similarly, oocytes are also deficient in carrying out glycolysis and cholesterol biosynthesis. For instance, denuded mouse oocytes can undergo maturation *in vitro* by providing pyruvate in the medium [50], [55]–[58], whereas oocytes co-cultured with cumulus cells mature in medium containing glucose as the only energy source [55]–[58]. These results indicated that oocytes cannot use glucose directly and thus require cumulus cells to provide the pyruvate metabolised from glucose for energy consumption by oocytes. In consideration of the location of CRCs and COCs, the cumulus cells convert the glucose into pyruvate, which the oocyte can utilise via direct transport through the gap junctions of the CRCs or via secretion by COCs and subsequent membrane transport [55]–[58]. In this study, the miRNAs were differentially expressed between CRCs and COCs, and after GO term annotation and pathway analysis we suggest that the energy substances supporting oocyte development and maturation might be primarily obtained from the production of CRCs under the regulation of miRNAs. Oocytes seem to lack the complete enzymatic system required for the synthesis of cholesterol, such as Mvk, Pmvk, Cyp51, Fbps, Sqle, and Ebp [56], [57]. In addition, the cholesterol receptors, e.g., SCARb1 and LDLR, are also not expressed in mouse oocytes. Furthermore, several studies also indicated that cholesterol from cumulus cells is the main source of oocyte cholesterol [55]–[58]. Our data suggested that miRNAs in the CRCs might be involved in cholesterol biosynthesis and the transport of cholesterol into the oocytes. In conclusion, oocytes undergo a prolonged and carefully regulated developmental process as a result of junctional interactions and instructive paracrine signalling with CRCs and COCs. The miRNAs seem to play a key role in the exchange of nutritional materials and regulatory signals between the oocytes and surrounding cumulus cells.

The immune system seems to regulate the development of the follicle and the corpus luteum, and its maintenance and regression, via the ovarian granulosa cells [59]. In this study, the differentially expressed miRNAs and novel miRNAs showed a strong over-representation of genes/pathways involved in immune regulation, e.g., T cell biology (GO: 0002361, CD4-positive, CD25-positive, alpha-beta regulatory T cell differentiation, Table 3; and GO:0031295: T cell costimulation, Table S3 and Table S4). Recently, NCOR1 was suggested to be involved in T cell biology (GO: 0002361; CD4-positive, CD25-positive, alpha-beta regulatory T cell differentiation, Table 3) [60]. Meanwhile, NCOR1 is also a component of the tamoxifen/oestrogen and receptor tyrosine kinase signalling pathway [61]. Furthermore, ovulation was found to be associated with tissue remodelling and inflammatory molecules at the site [59]. These findings suggested that miRNA-induced immunity regulation, such as the regulation of T cell biology, perhaps participates in ovarian cumulus cell-related processes.

In summary, for the first time we have analysed known and novel miRNAs in human stimulated preovulatory luteinizing CRCs and COCs by high-throughput Solexa sequencing. We have detected similarities and differences in the miRNA expression profile between CRCs and COCs, and confirmed their expression by quantitative real-time PCR analysis. The GO term annotation and KEGG pathway analysis for the predicted miRNA targets further indicate that these miRNAs are involved in various signalling pathways, such as amino acid and energy metabolism. Thus, the presence of a large number of miRNAs and the nature of their target genes suggested that miRNAs play important roles in the function of the follicular cumulus cells. Our work supports and further extends the knowledge of a regulatory role of miRNAs and their targeted pathways in folliculogenesis, which might facilitate the development of prophylactic strategies for the treatment of female infertility.

## Supporting Information

Figure S1 **Number of clean reads located on each chromosome in COCs and CRCs.**
(TIF)

Figure S2 **Number of miRNAs located on each chromosome in COCs and CRCs.**
(TIF)

Table S1 **The top 10 miRNA modifications in the miRNA expression profile of COCs and CRCs.**
(XLSX)

Table S2 **The top 10 miRNA editings in the miRNA expression profile of COCs and CRCs.**
(XLSX)

Table S3 **The differentially expressed miRNAs between COCs and CRCs.**
(XLSX)

Table S4 **GO analysis for predicted targets of novel miRNAs in CRCs.**
(XLSX)

Table S5 **GO analysis for predicted targets of novel miRNAs in COCs.**
(XLSX)

Table S6 **KEGG pathway analysis for predicted targets of novel miRNAs in CRCs.**
(XLSX)

Table S7 **KEGG pathway analysis for predicted targets of novel miRNAs in COCs.**
(XLSX)

File S1 **The miRNA primers used for quantitative real-time PCR.**
(XLSX)

File S2 **The putative target genes of the differentially expressed miRNAs.**
(XLSX)

File S3 **The novel miRNAs were identified in CRCs and COCs.**
(XLSX)
