# Supplementary figures and images for: Research Resources: Comparative MicroRNA Profiles in Human Corona Radiata Cells and Cumulus Oophorus Cells Detected by Next-Generation Small RNA Sequencing
Source: PLoS One. 2014 Sep 4;9(9):e106706. doi: 10.1371/journal.pone.0106706 (PMC4154750; doi:10.1371/journal.pone.0106706)

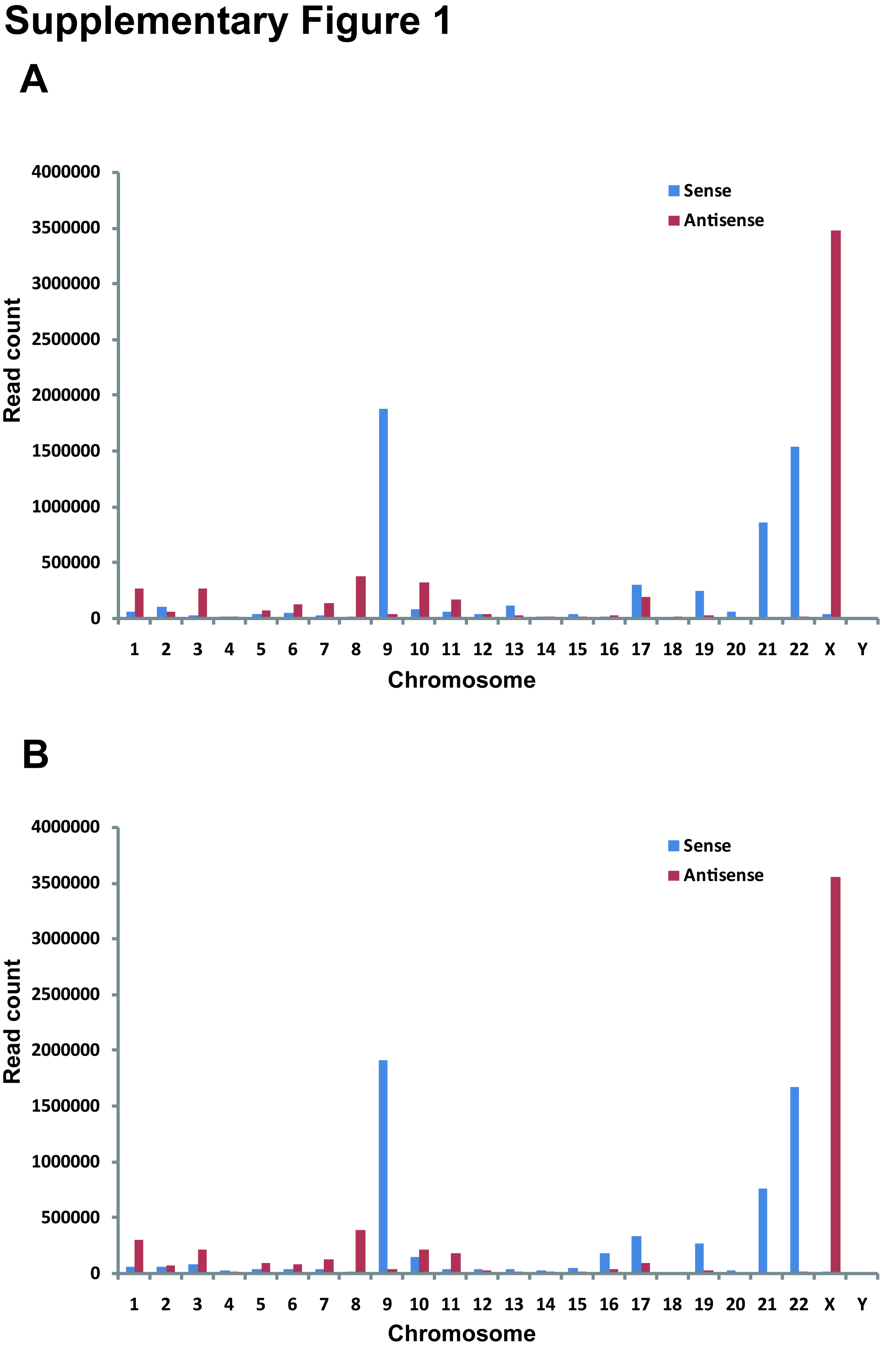

Supplement: Figure S1 — Number of clean reads located on each chromosome in COCs and CRCs. (TIF) [file pone.0106706.s001.tif]

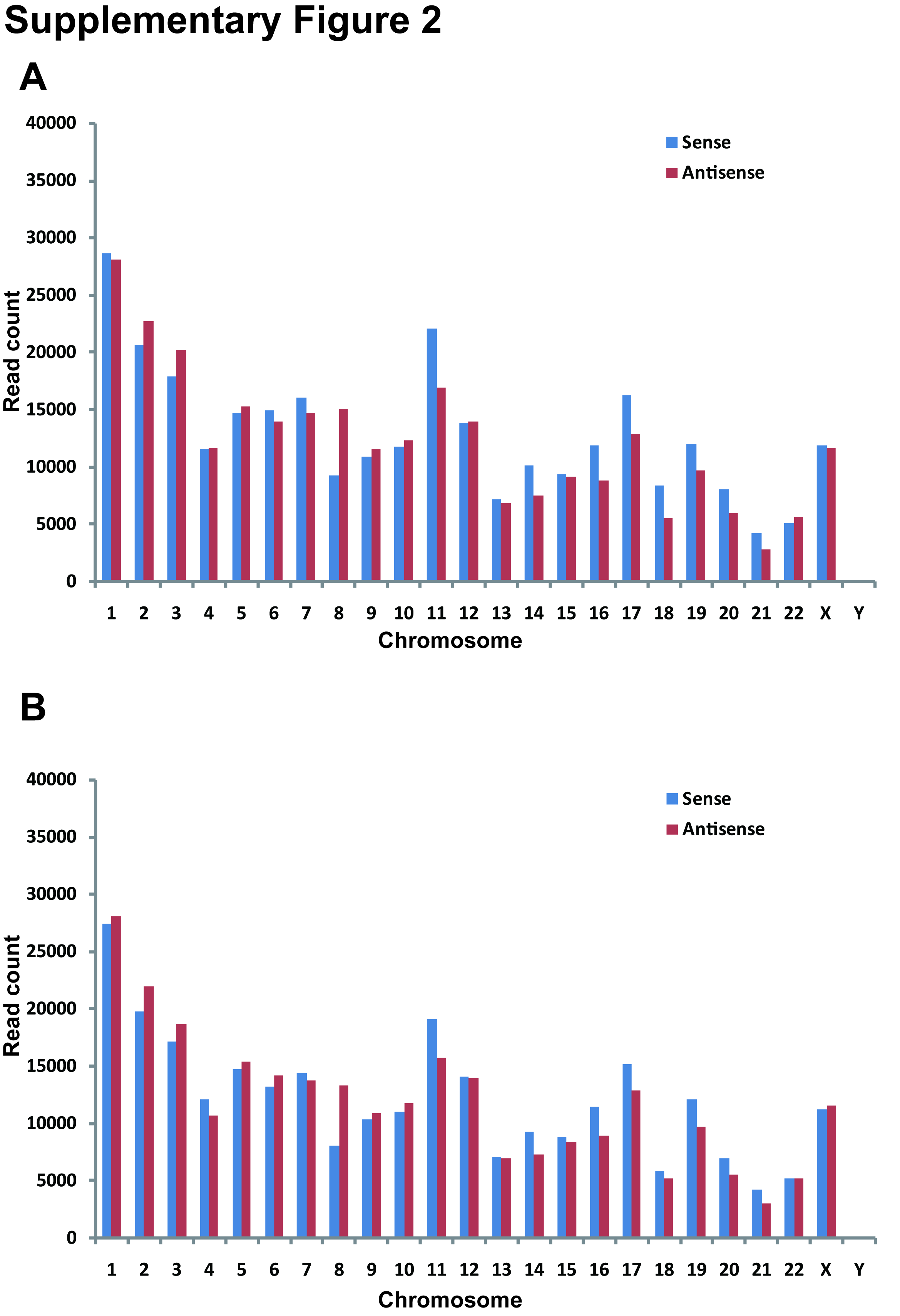

Supplement: Figure S2 — Number of miRNAs located on each chromosome in COCs and CRCs. (TIF) [file pone.0106706.s002.tif]
